# Historische Entwicklung der Diagnostik der bradykarden Rhythmusstörungen: Die frühen Jahre der Klinischen Elektrophysiologie in Deutschland

**DOI:** 10.1007/s00399-024-01006-0

**Published:** 2024-02-28

**Authors:** Günter Breithardt

**Affiliations:** Universitäres Herzzentrum, Klinik für Kardiologie, Universitätsklinikum Münster, Münster, Deutschland

**Keywords:** Bradyarrhythmie, His-Bündel-Elektrographie, Geschichte, Deutschland, Bradyarrhythmia, His bundle electrography, History, Germany

## Abstract

Die Einführung der His-Bündel-Elektrographie durch Benjamin Scherlag, New York 1969, zusammen mit der programmierten Stimulation des Herzens durch Philip Coumel, Paris 1967, und Hein Wellens, Amsterdam 1972, waren entscheidende Wendepunkte auf dem Weg zur invasiven Elektrophysiologie und der Entwicklung eines eigenständigen, inzwischen ausgeprägt interventionellen Schwerpunktes der Kardiologie. Das Hauptthema der 1970er Jahre waren die bradykarden Rhythmusstörungen, gefördert von der vor etwas mehr als 10 Jahren davor eingeführten Schrittmachertherapie. Die Ableitung der Potenziale des His-Bündels sowie weiterer Ableitungsorte in den Vorhöfen und Ventrikeln erlaubte eine differenzierte Beurteilung des Erregungsablaufs und der Refraktärzeiten. Die hochfrequente Vorhofstimulation zur Bestimmung der Sinusknotenerholungszeit und die vorzeitige Einzelstimulation zur Bestimmung der sinuatrialen Leitungszeit wurden zur Analyse der Sinusknotenfunktion entwickelt. In diesem Artikel wird die Einführung der His-Bündel-Elektrographie in einer allmählich zunehmenden Zahl von Zentren in Deutschland und ihr wissenschaftlicher Beitrag beschrieben.

## Abstract (en)

The introduction of His bundle electrography by Benjamin Scherlag (New York) in 1969, together with programmed stimulation of the heart by Philip Coumel (Paris) in 1967, and Hein Wellens (Amsterdam) in 1972, were decisive turning points on the way to invasive electrophysiology and the development of an independent, now distinctly interventional subspecialty of cardiology. The main topic of the 1970s was bradycardic arrhythmias, promoted by pacemaker therapy, which had been introduced just over 10 years earlier. The recording of the potentials of the bundle of His and other recording locations in the atria and ventricles allowed a differentiated assessment of the excitation process and the refractory periods. High-rate atrial stimulation to determine sinus node recovery time and premature stimulation to determine sinoatrial conduction time were developed to analyze sinoatrial node function. This article describes the introduction of His bundle electrography in a gradually increasing number of centers in Germany and their scientific contribution.

Die Einführung der His-Bündel-Elektrographie stellte einen Umbruch in der Diagnostik und Behandlung von Rhythmusstörungen dar. Aus dem anfangs *gemächlichen* Gebiet der Elektrokardiographie mit dem nicht zu verleugnenden Erkenntnisgewinn vieler Jahrzehnte wurde eine zunehmend interventionelle Subspezialität der Kardiologie.

## Entwicklung der His-Bündel-Elektrographie

Die Geschichte der Bradykardien während der letzten 50 bis 60 Jahre ist eng an die anfänglich lediglich als His-Bündel-Elektrographie bezeichnete neuartige Technik geknüpft, aus der sich im Laufe der Zeit eine Subdisziplin der Kardiologie, die Klinische Elektrophysiologie, entwickelte. Als Zeitzeuge und Mitwirkender während dieser Jahre liegt es nahe, sich auf das selbst Erlebte, aber auch das unmittelbar Vorhergehende, zu konzentrieren. Dabei verschwimmt die Erinnerung an die eigenen Studienjahre (1963–1968) in der Vielfalt des später erworbenen Wissens. Natürlich gehörte das EKG in der Physiologie und Pathophysiologie sowie in der Klinik zu den Lehrinhalten des Studiums. Auch lernten wir während des klinischen Studiums in Düsseldorf, gemäß dem Genius Loci, die wesentlichen Inhalte der 1961 noch pionierhaft, zunächst von Heinz-Joachim Sykosch und Sven Effert in Düsseldorf und kurz darauf von Paul Sunder-Plassmann in Münster, in Deutschland initiierten Schrittmachertherapie (siehe Artikel von B. Lemke in dieser Ausgabe 10.1007/s00399-024-01010-4).

Ein Blick in die eigenen Lehrbücher der Inneren Medizin aus jener Zeit zeigt, wie viel oder wie wenig damals über die Entstehung und die Erscheinungsform von Rhythmusstörungen bekannt war. Zu den aus meiner Sicht erwähnenswerten Lehrbücher, die sich speziell mit Rhythmusstörungen befassten, gehörten die von Eugen Lepeschkin [1], Hans Schäfer [2], Erich Schütz [3], Konrad Spang [4] und Max Holzmann [5].

Diese damaligen Standardbücher für den Spezialisten waren trotz des aus heutiger Sicht nicht zureichenden Wissens sehr umfangreich. Bereichert wurden diese Bücher durch ausführliche Kasuistiken und sehr instruktive EKG-Beispiele. Die von Rhythmusstörungen verursachte Symptomatik wurde oft breit erzählt, was auch heute oft noch lesenswert ist.

Viele Erklärungen der Pathogenese von Rhythmusstörungen waren unzureichend; es fehlte in vielen Fällen die heutige empirische Basis experimenteller, aber vor allem auch klinischer invasiver Untersuchungen. Dies sollte sich Anfang der 1970er Jahre mit Einführung der intrakardialen elektrographischen Ableitungen, der His-Bündel-Elektrographie und den hinzukommenden Stimulationsverfahren ändern.

### Im Fokus der 60er Jahre

Neue Konzepte und technische Durchbrüche spielen oft eine wesentliche Rolle bei der weiteren Entwicklung. So wurde das Interesse an den atrioventrikulären und intraventrikulären Blockierungen durch die Möglichkeit der Behandlung mittels implantierbarer Schrittmacher gefördert. Auf großes Interesse stießen die Arbeiten von Mauricio Rosenbaum aus Buenos Aires zur intraventrikulären Erregungsleitung und dem Konzept der Hemiblöcke [6, 7]. Darauf basierend hoffte man, bessere Kriterien im EKG zu entwickeln, um die Entstehung eines totalen AV-Blocks vorauszusagen. Mehrere Arbeitsgruppen haben sich hiermit in Deutschland Anfang der 70er Jahre beschäftigt (z. B. [8, 9]).

Auch die Pathoanatomie erlangte erneutes Interesse. Der Düsseldorfer Pathologe Hans-Jürgen Knieriem im Institut von Hubert Meessen, zusammen mit Sven Effert, später Aachen, der bei der ersten Schrittmacherimplantation in Düsseldorf mitgewirkt hatte, fasste 1966 die damaligen Befunde zur Morphologie des totalen AV-Blocks zusammen, gestützt auf das einschlägige Schrifttum und neuere eigene Untersuchungen [10]. Seine weiterführenden Untersuchungen erschienen 1974 in einer umfangreichen Monographie [11]. In ähnlicher Weise publizierte Lino Rossi aus Mailand über die Histopathologie der Arrhythmien [12].

**Figure 1:**
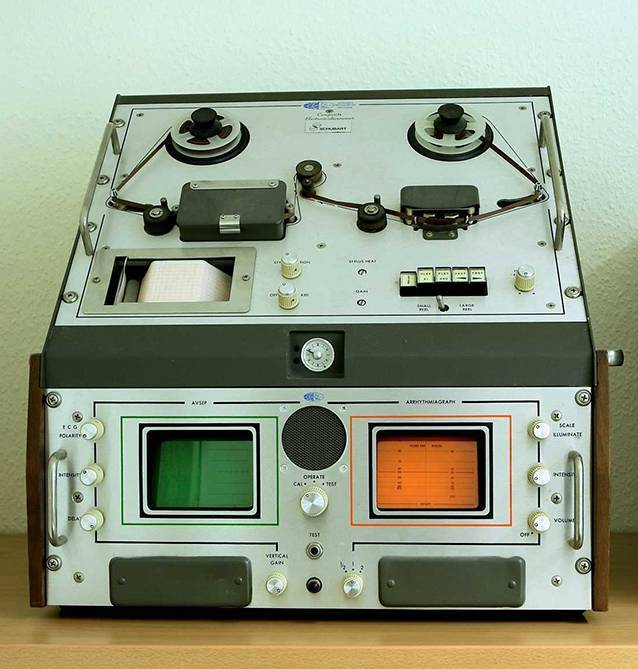

Eine technische Neuerung stellte die Einführung der kontinuierlichen EKG-Aufzeichnung dar. Diese Erfindung wird auch heute noch oft mit dem Namen von Norman Holter verbunden [13]. Das ursprünglich von Holter entwickelte Registriersystem musste in einem Rucksack getragen werden. Bereits 1965 kam das Holter Avionics^R^-System (Abb. 1) auf den Markt, dessen Aufzeichnungsgerät am Gürtel getragen werden konnte. Der Autor fand dieses Gerät (wahrscheinlich das Del Mar 650A) 1971 in der Loogen-Klinik in Düsseldorf vor, und machte daran bis in die späten Abendstunden seine ersten Erfahrungen in der Interpretation komplexer Rhythmusstörungen inklusive ihrer Abgrenzung zu Artefakten. Dieses System wurde mehrere Jahre lang klinisch benutzt. Hiermit waren EKG-Aufzeichnungen von 6 bis 8 h auf einer Tonbandspule möglich, die in einem RR-getriggerten Modus abgespielt wurden. Beim schnellen Abspielen des Bandes wurde bei weitgehend konstanten RR-Abständen ein konstant hohes Tonsignal und den RR-Intervallen entsprechend hohe Säulen auf dem Monitor erzeugt, aber bei vorzeitigen Schlägen oder bei Pausen änderte sich dann die Tonfrequenz, was sehr gut wahrnehmbar war, und die Länge der Säulen nahm ab oder zu. Man musste dann das Band stoppen, zurückspulen und dann 1:1 auf einem kleinen Monitor als EKG ansehen und evtl. ausschreiben.

Eine große Verbesserung der Langzeit-EKG-Technik stellte für uns der Reynolds Pathfinder dar, der in einem kleineren Aufnahmegerät im Vergleich zu Avionics Aufzeichnungen auf den damals handelsüblichen Tonbandkassetten bis zu 24 h ermöglichte. Zudem benutzte der Reynolds Pathfinder neben dem Audiosignal bereits komplexe Algorithmen zur QRS-Erkennung. Weitere Langzeit-EKG-Geräte folgten auf dem Markt. Bei einer der ersten Registrierungen in Düsseldorf mit dem Reynolds Pathfinder wurde bei einem Patienten in der Nacht der Beginn von Kammerflimmern registriert. Dieses Ereignis und natürlich die bereits damals bekannten Statistiken zur Häufigkeit des akuten Herztodes stimulierten das Interesse an der Risikostratifizierung und Prävention des akuten Herztods.

### Die 70er Jahre: das Aufkommen der invasiven Elektrophysiologie

**Figure 2:**
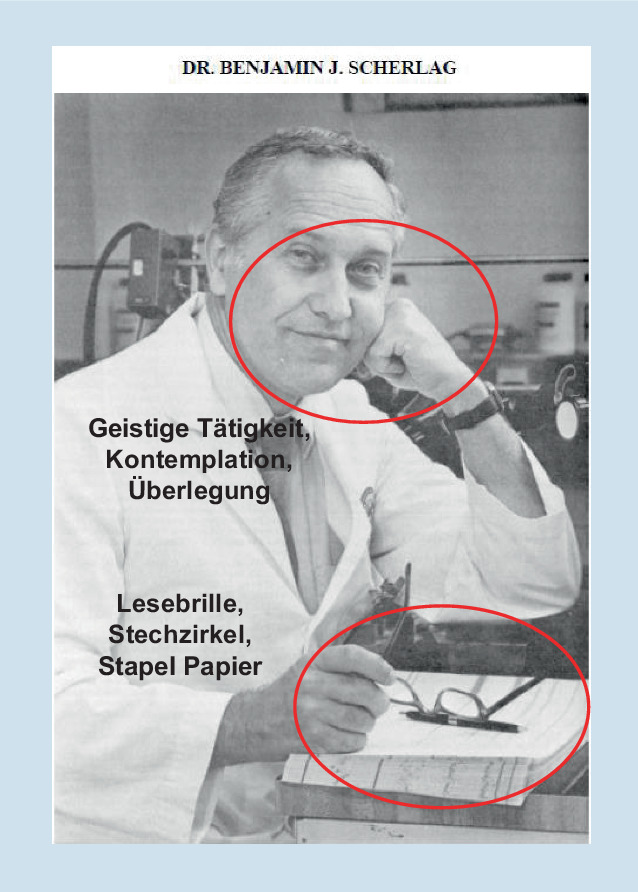

Die Möglichkeit der Schrittmachertherapie steigerte das Interesse am totalen AV-Block und möglichen Vorstufen. Dies wurde beflügelt durch die von Benjamin Scherlag (Abb. 2) und Mitarbeitern aus der Arbeitsgruppe von Anthony Damato 1969 als Routinemethode eingeführte Katheterableitung vom His-Bündel beim Menschen [14]. Einzelheiten aus der damaligen Zeit beschrieb Ben Scherlag kürzlich in Bezug auf seine eigene Krankheitsgeschichte [15].

Erste Registrierungen der elektrischen Potenziale des His-Bündels beim Menschen waren bereits von der Arbeitsgruppe von Paul Puëch, Montpellier, 1960 berichtet worden [16, 17]. Diese frühen Registrierungen erfolgten mit einem unipolaren, über eine Armvene eingeführten Katheter ohne Filterung der Signale, weshalb sich die Erfassung des Nutzsignals wegen starker Nulllinienschwankungen als schwierig herausstellte. Ähnliche Potenziale wurden von Watson et al. 1967 [18] registriert, offensichtlich auch mit einem unipolaren Katheter, aber wohl mit Hochpass-Filterung der Signale, wofür die stabile Grundlinie der Ableitung spricht. In der wegweisenden Veröffentlichung der Arbeitsgruppe von Anthony Damato und Benjamin Scherlag 1969 [14] wurde die Arbeit von Giraud, Puëch und Latour von 1960 [16] erwähnt.

Rückblickend war die Einführung der His-Bündel-Elektrographie zusammen mit der programmierten Stimulation des Herzens, inauguriert durch Philip Coumel, Paris [19] und Hein Wellens, Amsterdam [20], ein entscheidender Wendepunkt auf dem Weg zur invasiven Elektrophysiologie und der Entwicklung eines eigenständigen Schwerpunkts innerhalb der Kardiologie.

### Beginn der Klinischen Elektrophysiologie: ein persönlicher Rückblick

Motiviert durch die Veröffentlichung von Anthony Damato und Benjamin Scherlag [14] führte Ludger Seipel mit Unterstützung von Ulrich Gleichmann, später Bad Oeynhausen, die ersten His-Bündel-Ableitungen in Düsseldorf bei Patienten Ende Oktober 1971 durch. Ich durfte als junger Stationsarzt, der erst Anfang des Monats in der Klinik von Franz Loogen angefangen hatte, dabei sein und bei der Benutzung der noch einfachen Geräte helfen. Es sollten mehrere Untersuchungen bei weiteren Patienten notwendig sein, bis endlich ein sauberes His-Potenzial registriert wurde. Die Freude war groß (Heureka!).

Die ersten Veröffentlichungen aus der Düsseldorfer Arbeitsgruppe zeugen von dem zunehmenden Interesse an den Leitungsstörungen und speziell der Wirkung von Antiarrhythmika auf Leitungs- und Refraktärverhalten [21–23]. Bereits 1974 organisierte Ludger Seipel eine Arbeitstagung in Düsseldorf unter dem Titel „His-Bündel-Elektrographie“ [24]. 1978 kam dann sein Lehrbuch „His-Bündel-Elektrographie und intrakardiale Stimulation“ heraus [25]; 1987 kam die 2. neu bearbeitete und erweiterte Auflage hinzu [26]. An dieser Stelle seien auch zahlreichen Werke von Berndt Lüderitz [München, Bonn] genannt (z. B. [27, 28]), die ebenfalls eine große Verbreitung im deutschsprachigen Raum fanden.

Wir waren nicht die Ersten in der BRD, die Ableitungen vom His-Bündel anstrebten. Martin Schlepper und Helmut Neuss in der Kerckhoff Klinik in Bad Nauheim hatten als Erste in Deutschland mit der His-Bündel-Elektrographie begonnen. Gleichmann und Seipel hatten die Arbeitsgruppe in Bad Nauheim besucht, nachdem es ihnen bei Ableitungen beim Hund (am Ende von Experimenten in der Physiologie, mit anderer Fragestellung durchgeführt) nicht gelungen war, His-Bündel-Potenziale zu registrieren. Dagegen hatten Martin Schlepper und Helmut Neuss im Vertrauen auf die Veröffentlichung von Anthony Damato und Benjamin Scherlag unmittelbar beim Patienten diese Registrierungen durchgeführt.

Helmut Neuss, der zu Martin Schleppers wichtigstem Mitarbeiter bereits in der Frühzeit wurde, erinnert sich an diese Zeit [persönliche Mitteilung, Helmut Neuss, 03/2023]. Eigentlich sei er nur durch einen Zufall mit der Elektrophysiologie in Berührung gekommen. Nach 5 Jahren Weiterbildung am Niederrhein habe er eine Assistentenstelle an der Kerckhoff-Klinik angetreten. Martin Schlepper, Direktor der Kerckhoff-Klinik von 1972–1992, sei von einer Kardiologen-Tagung in den USA 1970 zurückgekommen mit der Publikation von Benjamin Scherlag über die His-Bündel-Elektrographie. „Das könnten wir auch hier machen.“ Martin Schlepper habe dann alle Hebel in Bewegung gesetzt, um die technischen Voraussetzungen zu schaffen. Er habe damals im Keller der alten Klinik den ersten Katheter geschoben und die ersten His-Signale aufgezeichnet, alle weiteren Untersuchungen habe er, Helmut Neuss, dann durchgeführt. Es gelang ihnen noch, den Abgabeschluss für die Frühjahrstagung der Gesellschaft für Kreislaufforschung einzuhalten und dort Befunde über die Auswirkungen von Verapamil auf die AV-Leitung vorzustellen [29]. So war an der Kerckhoff-Klinik, die damals eigentlich nur eine *bessere* Reha-Klinik gewesen sei, das Fundament für die sich entwickelnde Klinische Elektrophysiologie gelegt worden. Es dauerte nicht lange und Schlepper und Neuss bekamen den bereits erwähnten Besuch aus Düsseldorf – Ludger Seipel und Ulrich Gleichmann sahen sich ihre Registrierbedingungen an.

Die Anfänge der Klinischen Elektrophysiologie in Göttingen in der Arbeitsgruppe von Berndt Lüderitz mit Gerhard Steinbeck gingen ebenfalls auf 1971 zurück [persönliche Mitteilung, Gerhard Steinbeck, 2023]. Die intrakardialen Signale waren anfangs sehr störanfällig, so dass erkennbare His-Potenziale nur sporadisch gelungen seien; Publikationen über diese ersten Erfahrungen gebe es nicht. Das weitere Interesse von Herrn Lüderitz habe sich dann mehr der antitachykarden Stimulation als Therapieprinzip zugewandt, ausgelöst durch einen Besuch von Fred Zacouto aus Paris in Göttingen, der seinen orthorhythmischen Schrittmacher vorstellte.

Die Aachener Gruppe von Fleischmann und Pop benutzte für die Registrierung des His-Potenzials eine Bandpassfilterung von I00-270 Hertz. Die Signale wurden, wie in Göttingen, auf einem 8‑Kanal-UV-Schreiber oder auf einen ebenfalls achtkanaligen direkt schreibenden Düsenschreiber von Siemens (Erlangen) registriert [30]. Manfred Runge, der bei Onkar Narula in Miami gewesen war, kam mit dort gemachten Erfahrungen nach Hamburg zurück [31]. Erste Erfahrungen und Ergebnisse aus Mainz wurden 1973 berichtet in einer Übersichtsarbeit mit einigen eigenen Registrierungen, aber kleinen His-Potenzialen [32].

Weitgehend gleichzeitig erfolgten die Bemühungen in der DDR. Georg H. von Knorre, Rostock (persönliche Mitteilung, Georg H. von Knorre, 2023) und Mitarbeiter waren in Rostock die Ersten in der DDR, die sich mit der His-Bündel-Elektrographie beschäftigten. Ausgehend von der Publikation Scherlags [14] und den ihr folgenden Mitteilungen anderer Arbeitsgruppen sahen sie ebenso wie Schlepper und Neuss keine Notwendigkeit vorbereitender Tierversuche. Sie begannen ebenfalls 1971 und konzentrierten sich naturgemäß zunächst auf die atrioventrikulären und intraventrikulären Leitungsstörungen. Belegt ist dies durch ein Abstract eines Vortrags über retrograde Vorhoferregung beim AV-Block auf einer Veranstaltung 1971 in Italien [33], in der von Knorre Registrierungen mit Ableitung des His-Potenzials zeigte. Die erste reguläre Publikation zur His-Bündel-Elektrographie erschien 1972 [34]. Weitere Arbeiten folgten [35–37]. 1974 stieß Karl-Joachim Rostock zur Arbeitsgruppe, der rasch Gefallen an den damals aktuellen Untersuchungen zur gestörten Sinusknotenfunktion fand [38–40]. 1977 ging Karl Rostock nach Berlin an das Zentralinstitut für Herz-Kreislauf-Regulationsforschung der Akademie der Wissenschaften der DDR in Berlin-Buch, das die intrakardiale Elektrokardiographie in sein Repertoire aufnehmen wollte.

Ab der zweiten Hälfte der 1970er Jahre begannen auch andere Arbeitsgruppen in der DDR, die Methoden der intrakardialen Elektrokardiographie anzuwenden [persönliche Mitteilung, Georg Heinrich von Knorre, 2023]. Zu nennen seien Arbeitsgruppen an den Medizinischen Kliniken des Bezirkskrankenhauses Schwerin (Klaus Machill [41]), der Medizinischen Akademie Erfurt (Ingeborg Aßmann [42]), der Universitäten Leipzig (Annerose Neugebauer [43]) und Jena (Hans-Jürgen Volkmann [44]) sowie des Zentralinstituts für Herz-Kreislauf-Regulationsforschung der Akademie der Wissenschaften der DDR in Berlin-Buch (Karl-Joachim Rostock). Die Arbeitsgruppe von Karl Rostock in Berlin-Buch habe in den 1980er Jahren beachtliche Aktivitäten entwickelt. Darauf fußte schließlich auch Rostocks Buch „Herzrhythmusstörungen“ von 1988 [45]. Zu dieser Gruppe gehörten Karin Rathgen und Ulrike Krünes sowie Dietrich Pfeiffer und Alexander Schirdewan. Mitarbeiter der Charité Berlin begannen sich erst dafür zu interessieren, als in den 1980er Jahren die His-Bündelablation mittels Gleichstroms ihre kurze Karriere startete (Joachim Witte, Hans-Jürgen Bondke [46]). Vom Berlin-Bucher Kardiologen Hermann Fiehring und Karl Rostock ging ab 1979 schließlich auch die Initiative zu den Bad Liebensteiner Elektrokardiologischen Symposien aus, an deren 5. Veranstaltung im Januar 1988 der Verfasser teilnahm. Das sechste und letzte Symposium fand im Januar 1990 statt.

Die Erfahrungen an den einzelnen Einrichtungen mit den technischen und personellen Anfängen der elektrophysiologischen Arbeitsgruppen sind recht ähnlich gewesen. In allen jungen Arbeitsgruppen dürften diese Aktivitäten neben dem Mainstream der Tätigkeiten erfolgt sein, die im Vergleich zu heute noch recht beschränkt waren. Erst seit kurzem war in Deutschland die selektive Koronarographie eingeführt worden. Die sich für die junge Elektrophysiologie Begeisternden wurden als Außenseiter angesehen, die während der täglichen Arbeit die normalen klinischen Aufgaben zu erfüllen hatten, und dann in ihrer Freizeit dem *Hobby* nachgehen durften, das als kontemplativ und wenig handfest angesehen wurde.

### Apparative Ausstattung

Benjamin Scherlag und Mitarbeiter [14] beschrieben lediglich, dass der Wechselstromeingang eines EKG-Verstärkers benutzt wurde und dass die Signale mit Bandpassfiltern zwischen 0,1 und 200 Hz bzw. 40 bis 500 Hz registriert wurden. Dabei ist erstaunlich, dass selbst bei einer Hochpassfilter-Einstellung von 0,1 Hz noch eine gute Stabilität der Nulllinie erreicht wurde (Abb. 2 in ihrer Veröffentlichung). Zur (kontinuierlichen) Stimulation wurde ein einfacher externer Schrittmacher benutzt.

So einfach, nach heutigen Maßstäben primitiv, wie in der Veröffentlichung von Scherlag und Mitarbeitern [14], war auch in Deutschland die anfängliche apparative Ausstattung der klinisch-elektrophysiologischen Untersuchungsplätze. Schlepper und Neuss in Bad Nauheim erhielten Hilfe von Mitarbeitern des Physiologischen Instituts der Max-Planck-Gesellschaft, die ihnen z. B. aus Cournand-Kathetern Elektrodenkatheter bauten, ferner auch einen Schrittmacher entwarfen, mit dem sie eine programmierte Stimulation durchführen konnten. Das vorhandene Registriersystem der Klinik („Electronics for Medicine“) enthielt verschiedene Einschübe z. B. für Druck, EKG und auch für Phonokardiographie. Letzterer erlaubte eine optimale Filterung der elektrischen Signale, wobei insbesondere das Ausfiltern niedriger Frequenzen (unter 40 Hz) zu einer optimalen Darstellung der Signale führte [persönliche Mitteilung, Helmut Neuss, 03/2023].

Wir arbeiteten zunächst mit einem Tintendüsenschreiber von Siemens (Mingograph^R^), den wir uns jedes Mal aus der EKG-Abteilung leihen mussten. Erst später bekamen wir einen eigenen 6‑Kanal-Cardirex^R^-Schreiber von Siemens, der durch zwei Zusatzkanäle sowie einer Schalteinheit mit 50 Hz Hochpass-Filtern und Strombegrenzern ergänzt wurde. Alles wurde direkt auf Papier registriert, ein Fortschritt gegenüber den in manchen Ländern lange noch üblichen, umständlichen Registrierungen auf UV-Papier. Das Ergebnis waren über die Jahre anwachsende Papierstapel, die bis zu 500 oder mehr Seiten je nach Komplexität der Untersuchung umfassten. Zudem drang der Tintennebel bis in die Nasenlöcher des vor dem Schreiber sitzenden Registrierers (erst in späteren Jahren interessierten sich Kolleginnen für diese *seltsame* Spezialität). An das Tragen von Masken dachte keiner.

**Figure 3:**
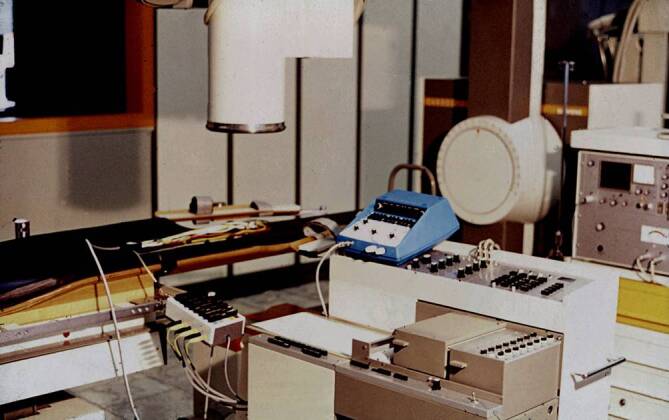

Als Stimulator diente uns ein nach unseren Wünschen modifizierter programmierbarer Stimulator Medtronic 5837. Dieses Gerät war ursprünglich von der Arbeitsgruppe Heinrich Kreuzer (später Stuttgart und Göttingen) und Bernhard Bostroem in Düsseldorf zur gepaarten Stimulation benutzt worden, um durch die induzierte postextrasystolische Pause über eine vermehrte diastolische Füllung eine positiv-inotrope Wirkung bei Herzinsuffizienz zu erzielen, was sich aber als der falsche Weg in der Behandlung des schlechten linken Ventrikels erwies. Später vewendeten wir einen programmierbaren Stimulator von Medtronic (Conduction System Analyser Model 5325) und noch später einen von Biotronik, bei dessen Entwicklung wir unsere Vorstellungen in der Handhabung einbringen konnten. Über mehrere Jahre führten wir unsere Untersuchungen auf einem zur Kardiologie gehörenden Durchleuchtungstisch durch, der uns erst am Nachmittag zur Verfügung stand. Mit Aufkommen der Ablationstechnik konnten wir auch den großen Herzkatheterraum der Klinik benutzen, aber erst 1984 beim Umzug in das neue Klinikgebäude bekam die Elektrophysiologie ein eigenes Herzkatheterlabor. Die frühe Entwicklung der Registriereinheiten zeigen Abb. 3 und 4.

**Figure 4:**
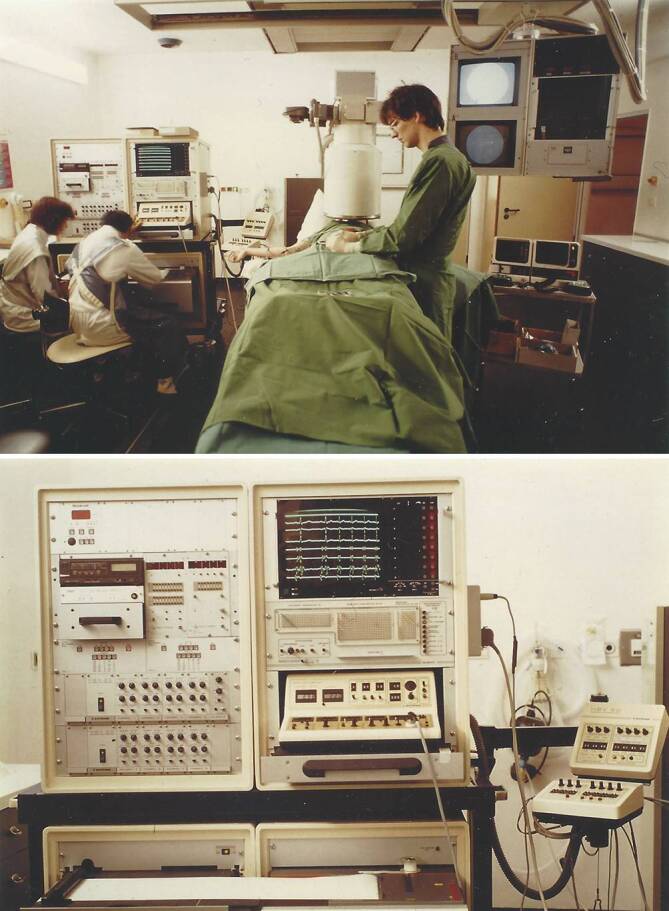

In Göttingen wurden für die intrakardialen Ableitungen und die Stimulation mit Hilfe eines Physikers aus einem DFG-Projekt ein Operationsverstärker (TektronixR Differenzverstärker Typ 3A3) und Stimulatoren von Tektronix aus dem physikalisch-technischen Bereich benutzt (Beschreibung in: [47]). Über eine Isolationseinheit (Tektronix Typ 2620) wurden Impulse von 2 ms Dauer mit doppelter Schwellenreizstromstärke bipolar erdfrei auf das distale Elektrodenpaar des Katheters abgegeben. Die Registrierung der Signale erfolgte auf einem UV-Schreiber (Fa. Hellige, Freiburg im Brsg, Deutschland).

Von Knorre berichtete weiter, dass mangels Beschaffbarkeit die Rostocker Arbeitsgruppe viel improvisieren musste. Ihre ersten His-Bündel-Elektrogramme schrieb sie mit einem für die hämodynamischen Registrierungen zum Katheter-Messplatz gehörenden Mingograph 81 (Elema-Schönander, Schweden). Über die mV-Eingänge konnte eine ausreichende Verstärkung der intrakardialen Elektrogramme erreicht werden. Es fehlte aber jegliche Möglichkeit zur Hochpassfilterung. Deswegen zeigten ihre ersten Publikationen ausschließlich ungefilterte Elektrogramme. Schließlich konnten sie die Einstellung eines Ingenieurs für die Abteilung durchsetzen, der ein zweikanaliges Filter als Zusatzgerät konstruierte [48]. Nach einem „Floating Input“ durfte dabei aber nicht gefragt werden. Mit ihrem eigenen 6‑Kanal-Direktschreiber-EKG-Gerät „6-NEK-4“ (Meßgerätewerk Zwönitz) aus DDR-Produktion gelangten so brauchbare Registrierungen, trotz eines *schönen* Kurven zuwiderlaufenden Kohlepapier-Durchschreibeverfahrens. Die Aufnahmen der Übersichtsarbeit „Die gestörte AV-Leitung im His-Bündel-Elektrogramm“ [49] sind alle damit registriert.

## Krankheitsbilder

Die Einführung der His-Bündel-Elektrographie markierte den Beginn der Klinischen Elektrophysiologie. Die schon seit einem Jahrzehnt bestehende Möglichkeit der Schrittmachertherapie und die neue Technik der His-Bündel-Elektrographie erklären die anfängliche Konzentration auf die Analyse der atrioventrikulären und intraventrikulären Erregungsleitung.

Die *reine* His-Bündel-Elektrographie mit Vermessung der AV-Überleitungszeiten wurde rasch ergänzt durch die zunehmende Verfügbarkeit von Stimulationsgeräten, die neben der einfachen hochfrequenten Stimulation auch die Abgabe vorzeitiger Impulse erlaubten. Hierdurch konnten nicht nur intrakardiale Leitungszeiten bei Spontanrhythmus oder unter hochfrequenter Stimulation gemessen werden, sondern durch die vorzeitige Impulsabgabe auch die Refraktärzeiten unter normalen und krankhaften Bedingungen ebenso wie vor und nach Gabe von Antiarrhythmika. Hinzu kam die Möglichkeit, die Sinusknotenfunktion durch hochfrequente Stimulation (Sinusknotenerholungszeit) oder durch programmierte vorzeitige Stimulation (sinuatriale Leitungszeit) zu analysieren.

### Atrioventrikuläre und intraventrikuläre Blockierungen

Die treibende Kraft in der Analyse und Bewertung intraventrikulärer und atrioventrikulärer Blockierungen ging von den Arbeitsgruppen in USA aus, während der Beitrag aus Deutschland anfänglich eher bescheiden war. Die hiesigen Untersuchungen trugen jedoch viel zum Aufbau klinisch-elektrophysiologischer Arbeitsgruppen bei, die sich in der Folgezeit vor allem den tachykarden Rhythmusstörungen zuwenden sollten.

Einige der Arbeitsgruppen sind bereits erwähnt worden. Neben der eigenen Arbeitsgruppe in Düsseldorf unter Leitung von Ludger Seipel seien hier genannt die Arbeitsgruppen in Aachen, insbesondere D. Fleischmann [30, 50, 55, 56, 96], in Mainz Klaus Lang und Hanjörg Just [32, 51, 52], in Bad Nauheim Helmut Neuss und Martin Schlepper [29, 53], in München Berndt Lüderitz und Gerhard Steinbeck [27, 28] und in Hamburg M. Runge [31, 54]. In der DDR gehörte die Arbeitsgruppe von Georg H. von Knorre in Rostock zu den Pionieren [33–37].

Das Interesse dieser Gruppen bezog sich einmal auf die prognostische Bedeutung von atrioventrikulären und intraventrikulären Leitungsstörungen im Hinblick auf die Entwicklung eines totalen AV-Blocks sowie die Erklärung seltenerer Phänomene wie das Lücken-Phänomen der atrioventrikulären Leitung (z. B. [30, 55]), atriale Leitungsstörungen [56] oder das Phänomen der verborgenen Erregungsleitung und verborgenen Extrasystolie im His-Bündel bei totalem AV-Block [36]. Hinzu kam ein zunehmendes Interesse, durch intrakardiale Ableitungen und Stimulationen die Wirkungscharakteristika von Antiarrhythmika besser zu verstehen, anfangs fokussiert auf Leitungsgeschwindigkeit und Refraktärität, erst wesentlich später mehr auf die Wirkung von Antiarrhythmika auf die Auslösbarkeit von Tachykardien. Besonderes Interesse zogen die intraventrikulären Leitungsstörungen (Schenkelblock und Hemiblöcke) auf sich im Hinblick auf die Möglichkeiten einer Schrittmachertherapie. Auf Grund retrospektiver Beobachtungen bei Patienten mit totalem AV-Block erschienen dabei insbesondere Patienten mit bifaszikulärem Block besonders gefährdet. Prospektive Studien ergaben jedoch, dass nur 1−2 % dieser Patienten einen höhergradigen AV-Block entwickelten [57–61]. Der gefürchtete akute Tod bei diesen Patienten war häufig auf tachykarde Rhythmusstörungen zurückzuführen [59, 62]. Serielle Untersuchungen belegten das Fortschreiten der Leitungsstörungen sowohl im Bereich der AV-Knotenleitung als auch infranodal [63]. Entscheidend für die Prognose war zudem die Grunderkrankung. Insbesondere Patienten mit verlängertem HV-Intervall hatten eine schlechtere Prognose auf Grund einer meistens fortgeschritteneren Grunderkrankung. So berichteten Dhingra und Mitarbeiter aus der Arbeitsgruppe von Kenneth Rosen in Chicago [64], dass elektrophysiologisch untersuchte Patienten mit chronischem Schenkelblock und einem HV-Intervall von 80 ms oder mehr nur dann eine hohe Morbidität und Letalität hatten, wenn eine symptomatische Herzerkrankung zu Grunde lag.

### Wirkung von Antiarrhythmika

Die Aufmerksamkeit auf Rhythmusstörungen stieg durch die Möglichkeit der ambulanten Erfassung mittels Langzeit-EKG, die sich ausbreitenden Intensivüberwachungs- und -therapiestationen sowie die Erkennung des Problems des akuten Herztodes. Man hoffte, durch intrakardiale Analyse die Mechanismen der Wirkung von Antiarrhythmika ebenso wie ihre Neigung, tachykarde oder bradykarde Arrhythmien auszulösen (Proarrhythmie), besser zu verstehen. Altbekannte, aber vielleicht in ihren Wirkungen und Nebenwirkungen nur eingeschränkt verstandene Antiarrhythmika [23, 65] wie Chinidin, Spartein, Procainamid, Phenytoin, Ajmalin, Lidocain, Betablocker [66], und erst seit kurzem Verapamil [29, 67, 68], wurden ergänzt durch zahlreiche neuere Antiarrhythmika wie Amiodaron, Propafenon [69], Flecainid [70], Aprindin [23], Disopyramid [69], Mexiletin, Encainid, Lorcainid, Moricicin, um die wichtigsten zu dieser Zeit zu nennen.

### Erkrankungen des Sinusknotens: Sick-Sinus-Syndrom, Sinusknotensyndrom, Sinusknotendysfunktion

Die Störungen der Sinusknotenfunktion hatten in früheren Jahrzehnten weniger Beachtung gefunden als die auf Grund ihrer Vielgestaltigkeit detaillierter bearbeiteten Störungen der AV-Überleitung und der intraventrikulären Leitung. Konrad Spang [4] beschreibt eine konstitutionelle Form der Sinusbradykardie bei offensichtlich gesundem Herzen mit unzureichendem Frequenzanstieg nach Atropin-Injektion. Zudem erwähnte er [4, p. 85] eine Beobachtung von Wenckebach, der ein familiäres Vorkommen in seiner eigenen Familie beobachtet hatte [72]. Wenckebach schrieb: „*In der Familie Wenckebach sind z.* *B. Vater, Mutter und 3 von 4 Kindern Bradykardiker. Ihre Pulsfrequenz fiel in der Jugend bisweilen unter 40; alle sind zu gesunden, kräftigen Menschen herangewachsen.“ *Unter Bezug auf Lewis beschreibt Spang [4] die von diesem so bezeichnete vagovasale Synkope [73]. Unter dem Stichwort „Toxische Sinusbradykardien“ [ibd., S. 86] beschreibt er, dass es im Rahmen einer Diphterie „in den ersten Krankheitstagen bei hochtoxischer Infektion zu einer hier wohl primär kardialen Sinusbradykardie kommen kann“.

Seine weiteren Ausführungen ebenso wie andere Werke lassen aber eine Beschreibung des Vollbildes der Sinusknotenerkrankung vermissen, wie es von Irene Ferrer 1968 als „Sick Sinus Syndrome in Atrial Disease“ in einem Übersichtsartikel, in dem sie drei Fälle beschrieb, zusammengefasst wurde [74]. Das von ihr beschriebene Syndrom (Sinusknotensyndrom; Syndrom des kranken Sinusknotens) umfasste persistierende, schwere und unerwartete Sinusbradykardien, Sistieren der Sinusknotenaktivität während kurzer Intervalle mit dazwischentretenden Ersatzrhythmen, lange Pausen von Sinusknotenstillstand ohne Ersatzrhythmen, unbehandeltes Vorhofflimmern mit langsamer Kammeraktion, kein Sinusrhythmus nach elektrischer Kardioversion und sinuatrialer Exitblock. Die Erfassung solcher, oft nur passager auftretender Störungen wurde in den vorhergehenden Jahren erst durch die neue Technik der Langzeit-EKG-Registrierung möglich. Aber nichts ist ohne Vorläufer. Bereits 1967 benutzten Bouvrain, Slama und Tekmine [75] den Begriff des „kranken Sinusknotens“. Zudem hatten R. J. Greenwood und D. Finkelstein bereits im Jahre 1964 die bis dahin bekanntgewordenen 223 Fälle in einer Monographie zusammengefasst [76]. Eine wahrscheinlich erste, größere Veröffentlichung aus dem deutschsprachigen Raum zum Sinusknotensyndrom erfolgte von DJ Athanasiou 1969 aus der Universitätsklinik in München [77]. Die Beobachtungsstudie umfasste 57 Patienten mit sinuatrialen Leitungsstörungen.

Einige Jahre später berichteten Alexander Wirtzfeld und H. Sebening [78], diesmal aus der Klinik der Technischen Universität München, über 29 Patienten mit bradykarden und tachykarden Vorhofarrhythmien, bei denen primär eine Störung der Sinusknotenfunktion vorlag mit Bradykardie, sinuatrialen Blockierungen oder einem Sinusstillstand, die ihrerseits das Auftreten ektopischer Tachyarrhythmien begünstigen. Die Symptomatik war vielgestaltig, der Verlauf unberechenbar. Jahrzehntelange weitgehende Beschwerdefreiheit trotz hochgradiger Bradykardie war bei ihren Patienten typischerweise ebenso anzutreffen wie intermittierende Asystolien mit schweren synkopalen Anfällen. Schon frühzeitig war es ersichtlich, dass eine medikamentöse Therapie meist nicht erfolgreich war, so dass sich häufig eine Schrittmacherindikation ergab, um nicht nur das Auftreten von Bradykardien und Asystolien zu verhindern, sondern um auch tachykarde Vorhofrhythmen wirksam zu unterdrücken.

Die Ursache des Krankheitsbildes war und ist auch heute noch weitgehend unbekannt, abgesehen vom familiären Hintergrund, wie schon bei Spang in der Familie Wenckebach beschrieben. Ebenso wie von Wirtzfeld und Sebening [78] berichtet, war auch von anderen Autoren eine Häufung von Fällen mit früherer Diphterie berichtet worden. Knut Rasmussen berichtete, dass 6 von 21 Patienten eine Diphterie durchgemacht hatten, 17 bis 48 Jahre vorher, im Mittel 30 Jahre [79]; bei Rolf Håkon Rokseth et al. [80] hatten 2 von 14 Patienten Diphterie gehabt, 3 weitere vorher rheumatisches Fieber. Im eigenen Kollektiv [81] fand sich eine Diphterie in der Vorgeschichte in 20,6 % der Fälle, was nach Auskunft des Gesundheitsamtes Düsseldorf wesentlich höher lag als die Erkrankungshäufigkeit in der allgemeinen Bevölkerung Jahre bis Jahrzehnte vorher.

Die sich zunehmend etablierende Schrittmachertherapie stellte den Anstoß dar, auch Sinusbradykardien in die differenzialdiagnostischen Überlegungen, z. B. bei Patienten mit Schwindelanfällen oder Synkopen, einzubeziehen. Gefühlsmäßig stellten sich damals mehr Patienten als heute mit dem vielgestaltigen Vollbild des Sinusknotensyndroms vor. Hat die weitgehende Beseitigung der Diphterie in unserer Bevölkerung zu diesem Wechsel im Krankheitsbild beigetragen?

Auf Grund klinischer sowie pathologisch-anatomischer Beobachtungen und insbesondere durch die His-Bündel-Elektrographie ließ sich zeigen, dass bei Patienten mit dem Sinusknotensyndrom nicht nur der Sinusknoten, sondern nicht selten auch der Atrioventrikularknoten („binodal disease“; [74]) und die distal gelegenen Abschnitte des Erregungsleitungssystems („panconductional defect“; [79]) in ihrer Funktion gestört sein können.

Unser eigenes Interesse an den Sinusknotenerkrankungen wurde geweckt, als der Verfasser auf einem Flug nach Wien zusammen mit seinem Mentor Ludger Seipel in der Ausgabe von *Circulation* vom Januar 1973 eine Veröffentlichung von Harold Strauss in Durham, North Carolina, las [82]. Die Vorstellung, auf die vorgeschlagene indirekte Weise eine Information zur sinuatrialen Leitungszeit und damit zu den klinischen Manifestationen wie sinuatriale Blockierungen und Sinusknotenstillstand zu erhalten, war stimulierend.

Das Prinzip des von Harold Strauss beschriebenen Verfahrens ist in Infobox 1 erläutert.

Die Beobachtungen der Gruppe um Harold Strauss stellte nicht nur für uns eine Aufforderung dar, diese Methode an einer größeren Zahl von Patienten zu untersuchen, sondern vor allem auch für Gerhard Steinbeck in der Arbeitsgruppe von Berndt Lüderitz. Die ersten Ergebnisse wurden 1974 von beiden Arbeitsgruppen in deutscher Sprache präsentiert und/oder veröffentlicht [47, 83, 84], später auch auf Englisch [85–87].

Eine Anekdote: Einer der Reviewer unserer Arbeit von 1977 in *Circulation* [86] monierte, dass wir eine alte Arbeit von Miki und Rothberger aus dem Jahre 1922 [88] zitiert hatten. Sie untersuchten die Länge der Pause nach vorzeitiger mechanischer Stimulation des Vorhofs und kamen bereits zu den gleichen Schlussfolgerungen wie Harold Strauss [82]. Wir wagten es, dem Reviewer zu widersprechen und drangen darauf, das Zitat im Text zu belassen. Dies war zu einer Zeit, in der wir in Deutschland noch nicht mit den Prinzipien eines Peer Reviews vertraut waren. Der Reviewer gab nach; dies stärkte unser Selbstwertgefühl.

Das Ergebnis dieser Untersuchungen war, dass Störungen der Sinusknotenfunktion, gemessen anhand spontaner Sinusbradykardie, verlängerter Sinusknotenerholungszeit und verlängerter sinuatrialer Leitungszeit, bei Patienten mit dem Sinusknotensyndrom oder mit spontanen sinuatrialem Block häufiger waren im Vergleich zu Patienten mit einer isolierten Sinusbradykardie [86]. Die Überlappungen der elektrophysiologischen Messwerte erlaubten jedoch keine für diagnostische Zwecke ausreichende Trennung, so dass für die klinische Anwendung die Beobachtung spontaner Bradykardien und längerer spontaner Pausen, z. B. im Langzeit-EKG, die entscheidende Größe im Hinblick auf die Schrittmacherindikation bildete.

Heftig wurden auf Tagungen die möglichen Ursachen für die unzureichende Aussagekraft elektrophysiologischer Parameter diskutiert. So beruht die Bestimmung der Sinusknotenerholungszeit auf einem komplexen Wechselspiel von Leitung und Impulsbildung im Sinusknoten [89]. Die Berechnung der sinuatrialen Leitungszeit aus der Länge der postextrasystolischen Pause beruhte auf der Annahme, dass während des postextrasystolischen Zyklus die Automatie des Sinusknotens sich nicht veränderte. Analysen der postextrasystolischen Zyklen sprachen aber für einen, zwar in der Regel, geringen depressiven Effekt der vorzeitigen Depolarisation auf die Zykluslänge des Sinusknotens, was jedoch zu einer Überschätzung der sinuatrialen Leitungszeit führen dürfte [85].

So können die eigenen Ergebnisse, die eine Verlängerung der postextrasystolischen Zyklen nach vorzeitiger Stimulation zeigten, damit erklärt werden, dass der erste Schlag nach der verlängerten poststimulatorischen Pause wegen der verlängerten Füllungszeit ein höheres Schlagvolumen hat, was über die Barorezeptoren zu einer Verlangsamung der Sinusknotendepolarisation führt. Die Geschwindigkeit dieses Refflexbogens war bereits von W. Delius, München, während seiner Zeit in Uppsala untersucht worden [90, 91].

Zudem ging die Berechnung nach dem Vorschlag von Harold Strauss [82] davon aus, dass retrograde und antegrade Leitungszeiten zwischen Vorhof und Sinusknoten identisch seien [85]. Gerhard Steinbeck, während seines Forschungsaufenthalts in der Arbeitsgruppe von Maurits Allessie in Maastricht, konnte in komplexen Mappinguntersuchungen am isolierten Vorhofpräparat von Kaninchen zeigen, dass die antegrade sinuatriale Leitungszeit länger ist als die retrograde; mit zunehmender Vorzeitigkeit des Stimulus nahm die retrograde Leitung um bis zu 5 ms zu. Die Bestimmung der sinuatrialen Leitungszeit im isolierten Präparat anhand der in der Klinik verwendeten Kriterien unterschätzte die gemessene Leitungszeit deutlich, wobei anzumerken ist, dass es im isolierten Präparat keine typische Plateauphase gibt [92]. Es darf aber nicht vergessen werden, dass sich das isolierte Sinusknoten-Vorhofpräparat von der In-situ-Situation unterscheidet, da die vegetative Innervation fehlt.

Dies ist etwas ausführlicher dargestellt, um die methodischen Probleme hervorzuheben, zu denen Arbeitsgruppen aus Deutschland international einen Beitrag geleistet haben. Andere Gruppen beschäftigten sich mit der Analyse der Sinusknotenautomatie [93–96]. Ferner haben Pop und Fleischmann sich früh für die atriale Vulnerabilität interessiert [71]. In der DDR fand Karl‑J. Rostock rasch Gefallen an den damals aktuellen Untersuchungen zur gestörten Sinusknotenfunktion. Hieraus resultierten einige Publikationen [38–40].

#### Sinusknotenstimulation und -ableitung.

Ein auffälliger, offensichtlich singulärer Befund war, dass über den gesamten Bereich der Vorzeitigkeit die postextrasystolischen Pausen konstant der spontanen Zyklusläge des Sinusrhythmus entsprachen. Wir konnten dies nur als [zufällige] Stimulation des wahrscheinlichen Zentrums des Sinusknotens interpretieren. Hieraus wurde eine antegrade Leitungszeit vom Sinusknoten auf den rechten Vorhof von etwa 50 ms gemessen [97]. Kritisch anzumerken ist, dass die postextrasystolischen Intervalle erst nach der Untersuchung vermessen wurden. Wäre dieser Befund während der Untersuchung bekannt gewesen und richtig interpretiert worden, hätte eine direkte Ableitung über die apikale, zur Stimulation verwendete Elektrode des mehrpoligen Katheters weitere Aufschlüsse geben können. Ähnliche Beobachtungen sind uns später nicht in der Literatur aufgefallen.

Bereits 1973 beschrieben bei intraatrialer Ableitung H.A. Warembourg et al. [98] atriale Potenziale, die kurz vor der P‑Welle lagen und die sie als Ausdruck der Sinusknotenaktivität interpretierten. Vinzenz Hombach et al. [99–101] setzten sich kritisch mit dieser Interpretation auseinander. In ihren eigenen Untersuchungen berichteten sie über 13 Patienten, bei denen sie eine Verifikation der vor der P‑Welle auftretenden Potenziale vornahmen. Auch berichteten sie von der Möglichkeit, mittels Signalmittelungstechnik über intraatriale Elektrodenkatheter Prä-P-Potenziale zu erfassen [102], wie erstmals von Stopczyk et al. beschrieben [103]. In komplexen experimentellen Untersuchungen haben R. Haberl und Mitarbeiter aus der Arbeitsgruppe von Gerd Steinbeck Probleme wie exakte Positionierung der Elektroden über dem Sinusknoten zur Bestimmung der sinuatrialen Leitungszeit herausarbeitet [104].

Unzureichend untersucht waren tachykarde Rhythmusstörungen, die auf einen Sinusknoten-Reentry zurückgeführt wurden. Ein nach heutigen Verhältnissen grobes Kathetermapping der Aktivierungssequenzen bei einem Fall, der die Kriterien des Sinusknoten-Reentry erfüllte, ergab, dass in der Tat die früheste Erregung im Bereich des Sinusknoten lag, was z. B. einen (seltenen) Reentry im Bachmann-Bündel ausschloss [105].

#### Wirkung von Antiarrhythmika.

Bekanntlich können Antiarrhythmika depressiv auf die Sinusknotenautomatie wirken. Ein typisches Beispiel ist Verapamil, das insbesondere bei Patienten mit Sinusknotensyndrom die Sinusknotenerholungszeit verlängern kann [67]. Während die Injektion von Verapamil bei normaler Sinusknotenfunktion reflektorisch über die periphere Vasodilatation zu einem Anstieg der Sinusknotenfrequenz führt, spricht der kranke Sinusknoten weniger oder gar nicht auf diese Änderung der vegetativen Innervation an; vielmehr wird hierbei die direkte negativ chronotrope Wirkung von Verapamil manifest. Hierfür sprechen die Befunde von Untersuchungen vor und nach autonomer Blockade mit Propranolol und Atropin [68]. Im Gegensatz dazu wies Flecainid eine deutlich geringere depressive Wirkung auf die Sinusknotenautomatie auf [70, 106, 107]. Nach Atropin-Injektion nahm die sinuatriale Leitungszeit [82] bei 11 Patienten ohne und mit Sinusknotendysfunktion ab, oder das Muster der postextrasystolischen Pausen normalisierte sich [108].

Bei der Bestimmung der Sinusknotenerholungszeit wird eine maximale Depression der Sinusknotenautomatie nur erreicht, wenn alle atrialen Erregungen den Sinusknoten erreichen. Die längste Sinusknotenerholungszeit wurde gewöhnlich bei niedrigerer Stimulationsfrequenz nach Gabe einer Substanz beobachtet, die die sinuatriale Leitungszeit verlängerte, während das umgekehrte Verhalten beobachtet wurde, wenn die sinuatriale Leitungszeit verkürzt wurde [109].

## Schlussfolgerung und Ausblick

Die 1969 von Scherlag und Mitarbeitern [14] inaugurierte klinische Methode der Ableitung von Potenzialen des His-Bündels war ein disruptives Moment, das tiefere Einsichten in die Erregungsausbreitung im menschlichen Herzen brachte. Im Laufe der 70er Jahre verbreitete sich die Methode nicht nur in Deutschland rasch und wurde zunehmend durch weitere Komponenten, z. B. elektrophysiologisches Mapping, ergänzt. Der nächste Schritt – weg von der reinen Diagnostik – waren Verfahren, um für die Entstehung von Rhythmusstörungen verantwortliche Strukturen auszuschalten, z. B. durch gezielte operative Verfahren (antitachykarde Chirurgie) oder mit Hilfe von Elektrodenkathetern (Ablation). Hinzu kamen die Möglichkeiten der Intervention in Form von antitachykarder Stimulation oder Kardioversion und Defibrillation mittels implantierter Geräte.

Heute, nach mehr als 50 Jahren, ist die Klinische Elektrophysiologie ein wichtiger Schwerpunkt innerhalb der Kardiologie.

### Prinzip des von Harold Strauss [82] beschriebenen Verfahrens zur Bestimmung der sinuatrialen Leitungszeit (Abbildung aus Seipel, L [25])


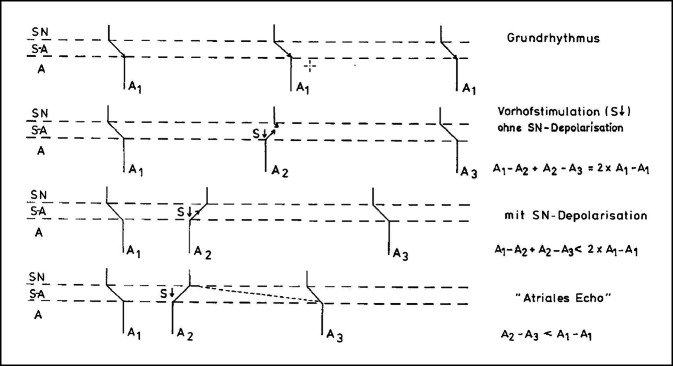


Stimuliert man den Vorhof vorzeitig, so kann die Erregung retrograd in den Sinusknoten eindringen, ihn zurücksetzen [„reset“], so dass er erneut einen Depolarisationszyklus beginnt; diese aus dem Sinusknoten kommende Erregung wird dann unter normalen Bedingungen in Richtung der Vorhöfe geleitet. Das Intervall der Vorhoftätigkeit nach dem vorzeitigen Stimulus bis zur nächsten spontanen Erregung setzt sich dann zusammen aus der retrograden Leitungszeit in den Sinusknoten, der Zyklusdauer der erneuten Depolarisation der Sinusknotens und der antegraden sinuatrialen Leitung. Die Summe der retrograden und antegraden Leitung zwischen Vorhöfen und Sinusknoten entspricht somit der Gesamtlänge der Post-Stimuluspause minus der spontanen Zyklusdauer des Sinusknotens. Dabei unterstellt man, dass die Zyklusdauer des Sinusknotens – nach der stimulusinduzierten Aktivierung – der spontanen Zyklusdauer vor dem Stimulus entspricht, was aber sicher nicht immer zutrifft. © Thieme, mit freundlicher Genehmigung. Diese Abbildung ist nicht unter der CC BY Lizenz veröffentlicht, sondern unterliegt Copyrightbeschränkungen
